# Predictive factors of extubation failure in pediatric cardiac intensive care unit: A single-center retrospective study from Thailand

**DOI:** 10.3389/fped.2023.1156263

**Published:** 2023-04-17

**Authors:** Kwannapas Saengsin, Rekwan Sittiwangkul, Thirasak Borisuthipandit, Konlawij Trongtrakul, Krittai Tanasombatkul, Thanaporn Phanacharoensawad, Guanoon Moonsawat, Phichayut Phinyo

**Affiliations:** ^1^ Division of Cardiology, Department of Pediatrics, Faculty of Medicine, Chiang Mai University, Chiang Mai, Thailand; ^2^ Division of Cardiology, Department of Pediatrics, Faculty of Medicine, Chiang Mai University, Chiang Mai, Thailand; ^3^ Division of Pulmonology and Critical Care, Department of Pediatrics, Faculty of Medicine, Chiang Mai University, Chiang Mai, Thailand; ^4^ Division of Pulmonary, Critical Care Medicine, and Allergy Department of Internal Medicine, Faculty of Medicine, Chiang Mai University, Chiang Mai, Thailand; ^5^ Center for Clinical Epidemiology and Clinical Statistics, Department of Family Medicine, Faculty of Medicine, Chiang Mai University, Chiang Mai, Thailand; ^6^ Faculty of Medicine, Chiang Mai University, Chiang Mai, Thailand

**Keywords:** extubation failure, pediatric cardiac patients, congenital heart disease, acquired heart disease, pediatric cardiac intensive care unit

## Abstract

**Introduction/objective:**

Extubation failure increases morbidity and mortality in pediatric cardiac patients, a unique population including those with congenital heart disease or acquired heart disease. This study aimed to evaluate the predictive factors of extubation failure in pediatric cardiac patients and to determine the association between extubation failure and clinical outcomes.

**Methods:**

We conducted a retrospective study in the pediatric cardiac intensive care unit (PCICU) of the Faculty of Medicine, Chiang Mai University, Chiang Mai, Thailand, from July 2016 to June 2021. Extubation failure was defined as the re-insertion of the endotracheal tube within 48 hours after extubation. Multivariable log-binomial regression with generalized estimating equations (GEE) was performed to explore the predictive factors associated with extubation failure.

**Results:**

We collected 318 extubation events from 246 patients. Of these, 35 (11%) events were extubation failures. In physiologic cyanosis, the extubation failure group had significantly higher SpO_2_ than the extubation success group (*P* < 0.001). The predictive factors associated with extubation failure included a history of pneumonia before extubation (RR 3.09, 95% CI 1.54–6.23, *P* = 0.002), stridor after extubation (RR 2.57, 95% CI 1.44–4.56, *P* = 0.001), history of re-intubation (RR 2.24, 95% CI 1.21–4.12, *P* = 0.009), and palliative surgery (RR 1.87, 95% CI 1.02–3.43, *P* = 0.043).

**Conclusion:**

Extubation failure was identified in 11% of extubation attempts in pediatric cardiac patients. The extubation failure was associated with a longer duration of PCICU stay but not with mortality. Patients with a history of pneumonia before extubation, history of re-intubation, post-operative palliative surgery, and post-extubation stridor should receive careful consideration before extubation and close monitoring afterward. Additionally, patients with physiologic cyanosis may require balanced circulation *via* regulated SpO_2_.

## Introduction

Pediatric Intensive Care Units (PICUs) frequently employ invasive mechanical ventilation (MV) as a life-saving intervention. The introduction of MV to treat respiratory failure in children has improved survival (1). Although MV is a cornerstone in critical care medicine, risks associated with prolonged MV include ventilator-associated pneumonia, ventilator-induced lung damage, and ventilator-induced diaphragmatic dysfunction (2). Premature extubation may become potentially problematic and result in emergent re-intubation with additional complications (3). Both premature and delayed extubation increase cost, morbidity, and mortality. MV should be discontinued as soon as the patient can maintain spontaneous breathing with appropriate gas exchange. Finding a proper time for extubation is important for intubated pediatric patients in ICU. Extubation failure in PICUs can considerably increase the risk of morbidity and mortality, with the incidence between 5% to 15% (4, 5). Several risk factors associated with extubation failure in pediatrics include young age, genetic syndrome, prolonged mechanical ventilation, duration of sedatives longer than five days, post-extubation stridor (PES), and decreased respiratory muscle strength (4, 5).

Even though recent studies have identified the factors associated with extubation failure in pediatrics, the knowledge on this topic is still limited in the pediatric cardiac population in both congenital and acquired heart diseases (6, 7). Prolonged open sternotomy, uncuffed endotracheal tube, the risk adjustment in congenital heart surgery-1 (RACHS-1), and PEEP greater than 5 mmHg are associated with extubation failure (8, 9). Abnormal distribution of pulmonary blood flow (Qp) and systemic blood flow (Qs) can occur with complex congenital heart disease. Thus, knowing the hemodynamics and optimizing Qp/Qs before extubation and other risk factors associated with extubation failure is crucial for intubated pediatric cardiac patients. These may decrease the incidence of extubation failure.

Our study aimed to 1.) describe the incidence of extubation failure in the Pediatric Cardiac Critical Care Unit (PCICU), 2.) identify predictive factors for extubation failure, and determine the association between extubation failure and clinical outcomes. This information could provide evidence to guide the development of future extubation protocols in this specific category of patients.

## Materials and methods

### Study design and population

We conducted a retrospective observational cohort study. The data on all extubation attempts in the PCICU of Chiang Mai University Hospital from July 2016 to June 2021 were retrieved and collected. The institutional review board of the Faculty of Medicine, Chiang Mai University, approved this study. We excluded patients aged less than one month and older than 18 years, tracheotomized patients, patients who had died under mechanical ventilation or following the withdrawal of support, those who were discharged with mechanical ventilation, and those who had undergone unplanned extubation.

Weaning was initiated by reducing the ventilator rate to below 20 breaths/minute and using low-level pressure support ventilation (PSV) with continuous positive airway pressure (CPAP). Pressure support was set to maintain a certain minimum tidal volume (TV = 4–6 ml/kg) and overcome endotracheal tube resistance for at least 30 minutes. The supported pressure was adjusted according to the diameter of ETT (ETT size 3.0–3.5 = PS of 10 cmH_2_O; ETT size 4.0–4.5 = PS of 8-9 cmH_2_O; ETT size 5.0 = PS of 6–7 cmH_2_O). We kept a minimum FiO_2_ of less than 40% with no acid-base disturbance and vital signs within the normal range for age. Patients with successful weaning in our center would be extubated.

### Data collection

We reviewed the patient's medical records and collected the following data: demographics, baseline clinical characteristics, history of cardiac surgery, risk adjustment in congenital heart surgery-1 (RACHS-1) score (9), history of intubation, and medical treatment during intubation. Vital signs and clinical conditions were collected within two hours before and after extubation. The laboratory data within the 72 hours before extubation were reviewed, and the data closest to the extubation time were selected. The MV parameters during extubation, including the size of the endotracheal tube (ET), ET tube cuffs, mode of the ventilator, peak inspiratory pressure (PIP), continuous positive airway pressure (CPAP), negative inspiratory force (NIF) (10), minute ventilation: V_E_ (L/min), and exhale tidal volume (V_Te_) were reviewed. Acute Physiology and Chronic Health Evaluation-II (APACHE-II) and pediatric risk of mortality III (PRISM III) scores at admission and before extubation were reviewed (11, 12). For the exploratory analysis, we pre-specified potential predictive factors that would be included in the multivariable analysis regardless of univariable statistical significance based on clinical importance and extensive review of clinical evidence as follows: age (4), genetic syndrome (5), history of pneumonia before extubation (13), and PES (7). The outcomes of this study included death and length of PCICU stay. All data were extracted from the electronic medical records and registered into the RedCap database (Vanderbilt University, Nashville, Tennessee).

### Definition

• Extubation failure was defined as re-intubation within 48 hours of extubation (1)
• Physiologic cyanosis was defined as the physiologic status of the patient during intubation, e.g., Tetralogy of Fallot (TOF) status post (s/*p*) modified right Blalock-Thomas-Taussig shunt (mRMBT), oxygen saturation should be less than 95%
• Physiologic acyanosis was defined as the physiologic status of the patient during intubation, e.g., Ventricular septal defect (VSD), TOF s/*p* total correction, oxygen saturation should be ≥ 95%
• Palliative surgery is defined as follows: a systemic to pulmonary artery (PA) shunt, a PA banding, a bidirectional cavopulmonary anastomosis, a Norwood operation, a Yasui operation, or a Nikaidoh operation
• History of re-intubation was defined as a re-intubation event during PCICU admission
• Pneumonia was defined as the occurrence of fever, productive sputum, the presentation of pulmonary infiltration in chest x-ray (CXR), and/or positive in sputum culture before extubation. History of pneumonia for whatever reason included community-acquired pneumonia (CAP), hospital-acquired pneumonia (HAP), and ventilator-associated pneumonia (VAP)
• PES was defined as 1) diagnosis of PES or clinical inspiratory stridor after extubation within 24 hours from medical record 2) medical administration of epinephrine nebulization, steroid nebulization, or intravenous after extubation within 24 hours
• High flow nasal cannula (HFNC) was defined as the use of a higher flow setting than inspiratory demand (the flow of 1–2 L/kg/min)

### Data analysis

Statistical analysis was performed using Stata 17 (StataCorp, College Station, Texas, USA). The statistically significant level was set at a *p*-value less than 0.05. Descriptive data were reported as frequencies and percentages for categorical variables and as mean and standard deviation (SD) or median and interquartile range (IQR) for numerical variables based on the underlying distribution. Demographic data, clinical characteristics, laboratory data, and MV parameters were compared among patients with extubation failure and patients with extubation success. The Pearson's chi-squared test was used to compare categorical data. However, the Fisher's-exact test was used when expected counts were <5. The t-test or Mann-Whitney *U* test was used to compare continuous variables based on their distribution.

In this study, the unit of observation was each extubation trial. Thus, one patient could be included within this dataset more than once. Multivariable log-binomial (risk ratio) regression was performed with a generalized estimating equations (GEE) to explore independent predictors for extubation failure. Exchangeable correlation or compound symmetric structure was specified for the GEE model. Prior to statistical modeling, all potential predictors were tested for collinearity; if positive for collinearity, only one variable deemed more important based on literature was included in the model.

Exploratory analysis was also conducted using the receiver operating characteristic curve (ROC). The ROC was used to identify the optimal oxygen saturation cut-off point in physiologic cyanosis that could predict extubation failure. Sensitivity, specificity, and area under the ROC curve (AuROC) were reported for each possible cut-off point.

## Results

### Study population

**Figure 1:**
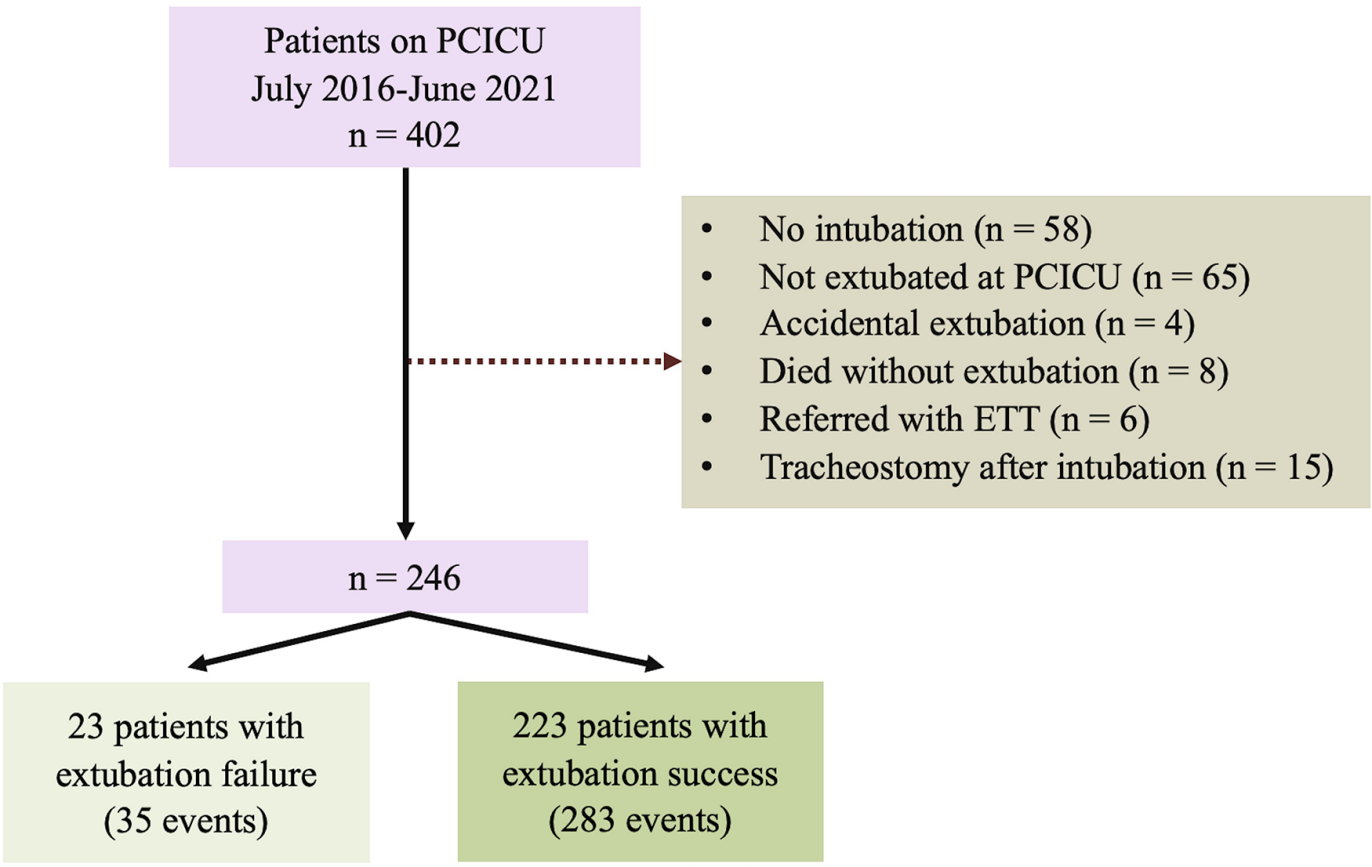
Flowchart of the study cohort. ETT; endotracheal tube; PCICU: pediatric cardiac intensive care unit.

**Figure 2:**
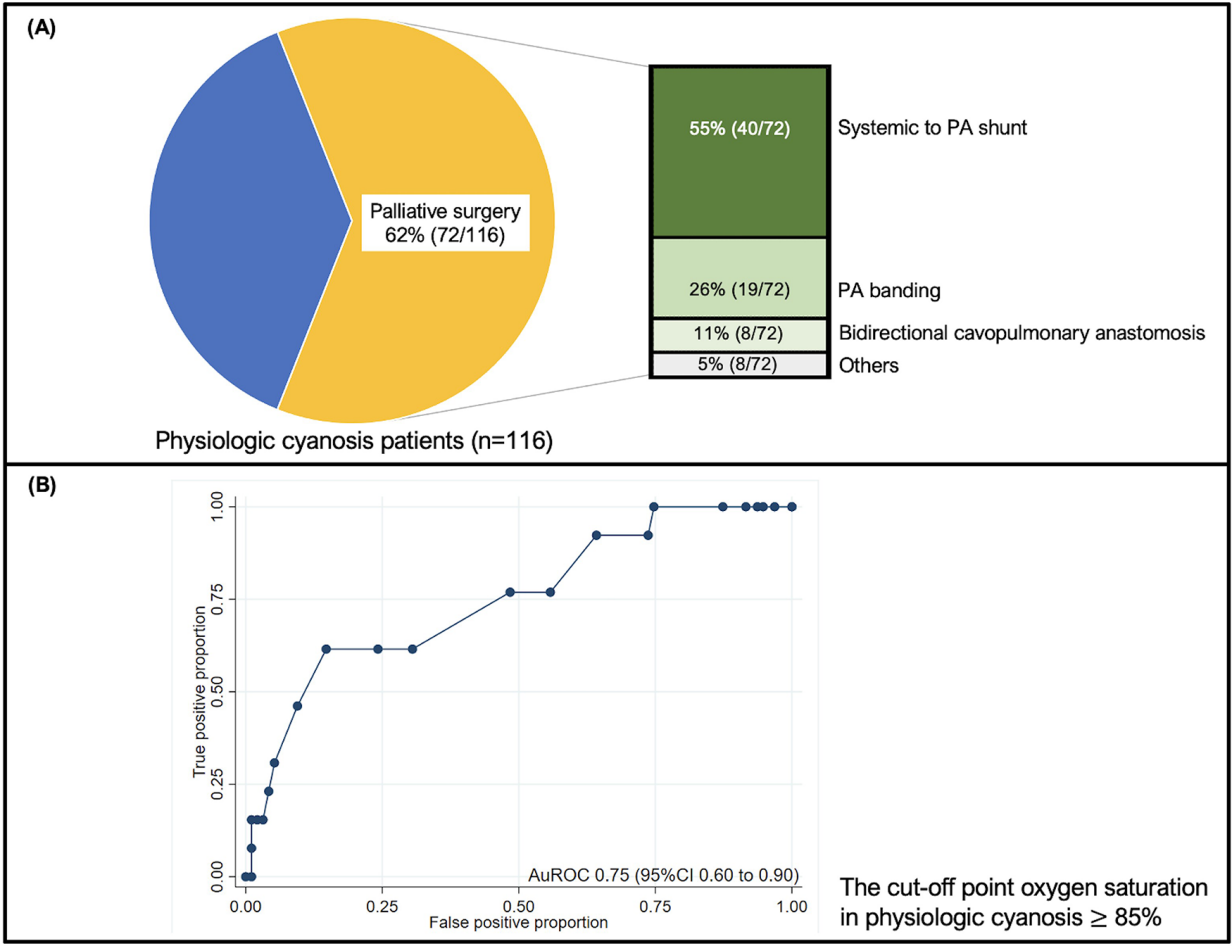
**(A)** The pie chart shows the proportion of patients with physiologic cyanosis who underwent palliative surgery. The number of patients who underwent surgery is presented in a bar graph; **(B)** The ROC graph illustrates the discrimination of SpO_2_ in predicting extubation failure in physiologic cyanosis. The cut-off point oxygen saturation in physiologic cyanosis  85%, and the sensitivity and specificity were 76.92% and 51.56%, respectively. The ROC curve shows 0.75 (95% CI 0.60–0.90). CI: confidence interval; PA: pulmonary artery; ROC: the receiver operating characteristic curve; SpO_2_: oxygen saturation.

In this cohort, there were 318 extubation events from 246 patients Figure 1. Of these, 35 (11%) events were extubation failures. No patients were re-intubated for a procedure. Demographic data, including gender, genetic syndrome, cardiac diagnosis, and cyanotic congenital heart disease, are reported in Table 1. Fifty percent of the patients were male. A genetic syndrome was found in 31.71% (78/246) of intubated patients. The three most common cardiac diagnoses were ventricular septal defect, functional single ventricle, and tetralogy of Fallot. Figure 2A depicts the proportion of patients with physiologic cyanosis who underwent palliative surgery.

**Table 1:** Baseline demographic and clinical data of all included patients at baseline of first extubation trial.

Variables	All patients (*N *= 246)	Extubation failure (*N *= 23)	Extubation success (*N *= 223)	*P*-value
Gender, male, *n* (%)	122 (49.56)	11 (47.83)	111 (49.78)	1.00
Genetic syndrome, *n* (%)	78 (31.71)	8 (34.78)	70 (31.39)	0.82
• Down syndrome	37 (47.44)	5 (50.00)	18 (25.71)	0.48
• Heterotaxy syndrome	20 (25.67)	2 (25.00)	18 (25.71)	
• 22q11 deletion syndrome	8 (10.26)	1 (12.50)	7 (10.00)	
• VACTERL association	3 (3.85)	1 (12.50)	2 (2.86)	
• Others	10 (12.82)	0 (0)	10 (14.29)	
**Primary cardiac diagnosis, *n* (%)**				
• Ventricular septal defect	52 (21.14)	4 (17.39)	48 (21.52)	0.55
• Functionally single ventricle	43 (17.48)	6 (26.09)	37 (16.59)	
• Tetralogy of Fallot	36 (14.63)	2 (8.70)	34 (15.25)	
• D-Transposition of great artery	16 (6.50)	1 (4.35)	15 (6.73)	
• Patent ductus arteriosus	13 (5.28)	2 (8.70)	11 (4.93)	
• Double outlet right ventricle	12 (4.88)	1 (4.35)	11 (4.93)	
• Atrioventricular septal defect	13 (5.28)	1 (4.35)	12 (5.38)	
• Coarctation of the aorta or interrupted aortic arch	9 (3.66)	1 (4.35)	8 (3.59)	
• Others	52 (21.14)	5 (21.74)	47 (21.08)	
Cyanotic congenital heart disease	120 (48.78)	11 (47.83)	109 (48.88)	1.00

Table 2 compares the baseline clinical characteristics for each extubation trial between successful and unsuccessful trials. BMI was significantly lower in the extubation failure group than in the extubation success group. The history of palliative surgery, closed heart surgery, and RACHS-1 risk category 3 were more common in the extubation failure group. The history of re-intubation and pneumonia was significantly higher in the extubation failure group than in the extubation success group. The respiratory rate was significantly higher in the extubation failure group than in the extubation success group. Supplementary Table S1 contrasts the difference in the remaining clinical characteristics between successful and unsuccessful trials. In physiologic cyanosis status, peripheral oxygen saturation (SpO_2_) was significantly higher in the extubation failure group and illustrated an acceptable discriminative performance at an AuROC of 0.75 (95% CI 0.60–0.90) (Figure 2B). The predictive indices of oxygen saturation in physiologic cyanosis to extubation failure cut-off point are shown in Supplementary Table S2. At the 85% oxygen saturation cut-off point in physiologic cyanosis, the sensitivity and specificity were 76.92% and 51.56%, respectively. APACHE II scores before extubation were significantly higher in the extubation failure group. PES, the required adrenaline nebulizer, and high-flow nasal cannula after extubation were more common in the extubation failure group.

**Table 2:** Comparison of clinical parameters for each included extubation trial between successful and unsuccessful trials.

Variable	Extubation failure (*N* = 35)	Extubation success (*N* = 283)	*P*-value
**History of intubation and medical treatment before extubation**			
Age, months (median, IQR)	7.27 (1.93,22.13)	9.69 (2.93,20.79)	0.36
– Infant (30d to 1 yr), *n* (%)	22 (62.86)	160 (56.54)	0.59
– Child (1–18 yr)	13 (37.14)	123 (43,46)	
Weight, kg (median, IQR)	5 (3.5,8)	5.8 (3.9,9.3)	0.27
Height, cm (median, IQR)	61 (54,75.5)	65 (55,78)	0.60
BMI, mean (SD)	12.86 (2.94)	14.18 (3.13)	**0**.**019**
Post-surgery, *n* (%)	20 (57.14)	168 (59.36)	0.86
• Palliative surgery	14 (70.00)	58 (34.73)	**0**.**003**
• Total correction	6 (30.00)	109 (65.27)	
**Closed vs. open surgery, *n* (%)**			
• Closed surgery	16 (80.00)	64 (38.10)	**0**.**001**
• Open surgery	4 (20.00)	104 (61.90)	
**Procedure by risk category (RACHS-1), *n* (%)**			
• 1	0 (0)	11 (6.5)	**0**.**049**
• 2	3 (15.00)	61 (36.31)	
• 3	17 (85)	80 (47.62)	
• ≥4	0 (0)	14 (9.52)	
Post-intervention, *n* (%)	0 (0)	4 (1.41)	1.00
Duration of intubation, days (median, IQR)	6 (4,12)	6 (3,11)	0.26
History of re-intubation in admission, *n* (%)	6 (17.14)	13 (4.59)	**0**.**029**
VIS before extubation 48 hours (median, IQR)	0 (0,2)	0 (0,2)	0.89
Steroid before extubation, *n* (%)	19 (54.29)	139 (49.12)	0.59
Continuous Sedation before extubation 48 hours, *n* (%)	10 (32.26)	87 (41.63)	0.43
Duration of fentanyl, days (median, IQR)	0 (0,2)	1 (0,3)	0.38
Duration of midazolam, days (median, IQR)	0 (0,0)	0 (0,2)	0.13
Duration of morphine, days (median, IQR)	0 (0,0)	0 (0,0)	0.58
Muscle relaxant, *n* (%)	2 (5.71)	38 (13.43)	0.28
Duration of muscle relaxant (days)	0 (0,0)	0 (0,0)	0.21
CXR atelectasis before extubation, *n* (%)	2 (5.71)	10 (3.53)	0.63
Pneumonia, *n* (%)	26 (74.29)	120 (42.40)	**<0**.**001**
Pulmonary hypertension, *n* (%)	7 (20.00)	98 (34.630	0.09
**Vital signs and clinical condition before extubation**			
Respiratory rate (RR), cycles/min, mean (SD)	40 (10)	37 (9)	**0**.**036**
Heart rate (HR), beats/min, mean (SD)	125 (18)	119 (20)	0.10
Body temperature (BT), Celsius, mean (SD)	36.88 (0.45)	37.12 (3.69)	0.82
Systolic blood pressure (SBP), mmHg, mean (SD)	93 (17)	95 (13)	0.39
Diastolic blood pressure (DBP), mmHg, mean (SD)	55 (14)	36 (12)	0.69
Mean arterial blood pressure (MAP), mmHg, mean (SD)	68 (13)	69 (11)	0.55
SpO_2_ (%), (median, IQR)	94 (88,100)	97 (86,100)	0.57
• Physiologic cyanosis	88 (4)	83 (4)	**0**.**001**
• Physiologic acyanosis	98 (3)	98 (3)	0.85
GCS, mean (SD)	10 T (0)	10 T (0.2)	0.41
APACHE II before extubation, mean (SD)	11.25 (4.06)	9.57 (3.25)	**0**.**04**
PRISM III before extubation, mean (SD)	4 (0,7)	2 (0,4)	0.09
**Clinical condition, and management after extubation**			
Post extubation stridor, *n* (%)	19 (54.29)	70 (24.73)	**0**.**001**
Adrenaline nebulizer after extubation, *n* (%)	19 (54.29)	71 (25.09)	**0**.**001**
Steroid after extubation, *n* (%)	16 (45.71)	111 (39.22)	0.47
High flow nasal cannula after extubation, *n* (%)	27 (77.14)	89 (31.45)	**< 0** **.** **001**

APACHE, Acute physiology and chronic health evaluation; CXR, chest x-ray; GCS, Glasgow Coma Scale; IQR, inter-quartile range PRISM, pediatric risk of mortality; RACH-1, the risk adjustment in congenital heart surgery-1; VIS, Vasoactive-Inotropic Score.

### Predictive factors associated with extubation failure

All the pre-specified predictive factors showed statistical significance in the univariable analysis, except for age and genetic syndrome. In multivariable analysis, the independent predictive factors for extubation failure were a history of pneumonia before extubation (RR 3.09, 95% CI 1.21–4.12, *p* = 0.009), PES (2.57, 95% CI 1.44–4.56, *p* = 0.001), history of re-intubation (RR 2.24, 95% CI 1.21–4.12, *p* = 0.009), and palliative surgery (RR 1.87, 95% CI 1.02–3.43, *p* = 0.043) (Table 3). Age, genetic syndrome, and BMI did not significantly affect extubation failure.

**Table 3:** Results from the multivariable log-binomial regression with generalized estimating equations exploring predictive factors associated with extubation failure.

Variable	Risk ratio	95% CI	*P*-value
Age at extubation, month	1.00	0.99–1.01	0.266
BMI	0.88	0.74–1.04	0.412
Pneumonia	3.09	1.54–6.23	0.002
History of re-intubation	2.24	1.21–4.12	0.009
Palliative surgery	1.87	1.02–3.43	0.043
Post extubation stridor	2.57	1.44–4.56	0.001
Genetic syndrome	1.53	0.78–3.01	0.216

BMI, body mass index; CI, confidence interval.

### Association of extubation failure with patient outcomes

There is no statistical difference between extubation failure and success groups regarding the incidence of death (8.57% vs. 3.18%, *p* = 0.134). Duration of PCICU stay was significantly longer in the extubation failure group (median: 26 days vs. 7 days; *p* < 0.001).

## Discussion

This study reported that extubation failure in PCICU occurred in 11% of extubation events. The predictive factors associated with extubation failure included the diagnosis of pneumonia before extubation, history of re-intubation, underwent palliative surgery, and stridor after extubation. The extubation failure was associated with a longer duration of PCICU stay but not with mortality. Additionally, in pediatric cardiac patients with physiologic cyanosis, the extubation failure group had significantly higher SpO_2_ than the extubation success group. Our study is consistent with previous studies. Extubation failure in the pediatric ICU ranges from 5% to 15% (4, 5). Our findings align with the previous literature regarding extubation failure being associated with longer ICU stays (5). As BMI is a simple and standard measure to assess nutritional status (14), a previous study showed malnutrition was an independent association with a longer length of mechanical ventilation usage (15). Our study showed that BMI was significantly lower in the extubation failure group. The APACHE-II score is one of the measurement tools used to evaluate the illness severity of PICU and PCICU patients (15, 16). It has also been a potential predictive factor in adult extubation failure (13, 15, 17). Our study demonstrated that the APACHE II score was significantly higher in the extubation failure group than in the extubation success group. Therefore, the APACHE II score may be applied as a predictive factor for extubation failure in pediatric patients, as it is in adults.

In the extubation failure group, palliative surgeries, including PA banding (57%), systemic to PA shunt (36%), and bidirectional cavopulmonary anastomosis (7%), were predictive factors associated with extubation failure. J.W. Miller et al. reported that procedural complexity was associated with extubation failure (18). The incidences of extubation failure were presented as follows, systemic to PA shunt (67%), PA banding (50%), and bidirectional cavopulmonary anastomosis (14%) (18, 19). Closed heart surgery and RACSH-1 category 3 were common in the extubation failure group. We found that 81% of closed heart surgeries and most of the RACSH-1 category 3 patients were palliative surgeries that might be collineated. This evidence may emphasize that palliative surgery could be one of the predictive factors of extubation failure.

The physiologic cyanosis group with mixing lesions included patients who underwent palliative surgery, where Qp vs. Qs was determined by pulmonary vascular resistance (PVR). Because oxygen is one of the potent pulmonary vasodilators which decreases PVR, the previous study showed an increase in pulmonary blood flow during high concentrations of oxygen (hyperoxia) (20). Moreover, The increased pulmonary blood flow is related to increased ventricle volume load and decreased systemic circulation, which may be associated with extubation failure (21–24). Arterial oxygen saturation (SaO_2_) has become the target for balancing pulmonary blood flow and systemic circulation. SaO_2_ of 75%–80% is believed to reflect a balanced circulation with Qp/Qs of 1 (21). Due to the good correlation between SaO_2_ and SpO_2_, SpO_2_ is a simple, non-invasive method to estimate SaO_2_ (25). In our study, we recommend not keeping high levels of SpO_2_. Controlling SpO_2_ around 75%–85% in patients with physiologic cyanosis by adjusted FiO_2_ is recommended to achieve a balanced pulmonary blood flow and to optimize systemic oxygen delivery. The high SpO_2_, despite optimum adjusted FiO_2_, may lead to increased pulmonary blood flow. Therefore, we recommend adjusting PEEP, allowing mild hypoventilation to increase PaCO_2_ to the normal upper limit, and balancing fluid intake output to control pulmonary blood flow before extubation (22).

Pneumonia causing inflammation in the lung results in reduced gas exchange and increases the work of breathing (26). Narrowing of the airway can result from infection-related mucosal edema and secretory outbursts that frequently become physiologically relevant at the level of the small airway obstruction (27). Abnormal lung function may be one of the risk factors for extubation failure. The respiratory rate will increase in response to a decreased tidal lung volume, causing falling oxygen levels and rising carbon dioxide levels (26). Our study is consistent with previous studies (13).

Re-intubation can damage the mucosa, causing shearing, airway trauma, and edema, which may induce post-extubation stridor (PES). Re-intubation may increase the total time of intubation in the PCICU. Previous studies reported prolonged intubation as a risk factor associated with extubation failure (5, 28).

PES may arise from laryngeal edema and mechanical stress to the larynx (29). Airflow through a narrowed upper airway presents clinically as PES, the most common reason for high respiratory effort. Patients with PES are likely to have increased incidences of re-intubation (30–32). Khemani et al. reported that the re-intubation rate of patients with PES was 47.4% and 5.7 times higher than the population average (7). In pediatrics with cardiac disease, escalating airway resistance could alter the limited cardiopulmonary reserve and increase ventricular wall stress (8). This finding emphasizes the importance of close monitoring for respiratory effort after extubation. The therapeutic management for PES includes adrenaline nebulization after extubation, systemic steroid administration for reducing airway inflammation, or an application of HFNC for reducing anatomical dead space, decreasing subglottic laryngeal inflammation, and reducing airway resistance, which appears to have assisted in avoiding re-intubation (33–35). Not surprisingly, in our study, adrenaline nebulized after extubation, systemic steroid administration, and HFNC use were found more often in the extubation failure group, probably from the higher incidence of PES in the extubation failure group.

## Study limitations

Our study has some limitations. First, the data used were retrospectively collected based on the availability of the data. Thus, some factors could not be gathered at the exact time point in all patients, and a number of potential predictive factors could not be examined, such as the duration of surgery and the occurrence of post-operative cardiac output syndrome. In addition, the routine protocol for weaning and extubation during the study period might not be sufficiently controlled as in trials or prospective studies. In our center, we found that the weaning protocol was used in more than 80% of extubation events. Second, this was a single-center study, which would limit the generalizability of our results to other clinical contexts with different backgrounds. Third, as our center only admits children older than one month to the PCICU, neonatal patients admitted to the neonatal intensive care units (NICU) were not included. Fourth, the sample size in this study was limited owing to the low prevalence of the domain of interest. Future multicenter studies with more extubation failures may improve the statistical power to identify additional significant risk factors. Lastly, the duration for defining extubation failure varied between 24 and 48 hours. The associated predictors of extubation failure may be different according to each definition. In our study, we defined extubation failure as 48 hours after extubation, which is used in most reviewed publications.

## Conclusion

Our analysis suggests that extubation failure in the PCICU still matters and was associated with an increased PCICU stay. Assessment of predictive factors of extubation failure may be an opportunity to improve extubation success rates. Patients diagnosed with pneumonia, history of re-intubation, post-operative palliative surgery, and post-extubation stridor should receive careful consideration before extubation and close monitoring afterward. Patients with physiologic cyanosis may also require balanced circulation *via* regulated SpO_2_.

## Data Availability

The raw data supporting the conclusions of this article will be made available by the authors, without undue reservation.
