# Supplementary material for: Predictive factors of extubation failure in pediatric cardiac intensive care unit: A single-center retrospective study from Thailand
Source: Front Pediatr. 2023 Apr 17;11:1156263. doi: 10.3389/fped.2023.1156263 (PMC10149922; doi:10.3389/fped.2023.1156263)
Supplement: Supplementary file 1 [file Table1.docx]

**Supplementary Table S1:** Comparison of the remaining clinical parameters for each included extubation trial between successful and unsuccessful trials

| **Variable** | **Extubation**  **Failure**  **(N=35)** | **Extubation**  **Success**  **(N=283)** | **P-value** |
| --- | --- | --- | --- |
| ***Laboratory values within 36 hours before extubation*** | | | |
| Venous blood gas |  |  |  |
| - pH, mean (SD) | 7.42 (0.54) | 7.42 (0.07) | 0.43 |
| - pCO_2_, mmHg, mean (SD) | 46.12 (8.93) | 48.11 (9.33) | 0.35 |
| - Bicarbonate, mmol/L, mean (SD) | 29.85 (5.04) | 30.49 (5.99) | 0.62 |
| - Venous oxygen saturation (SvO_2_), %, mean (SD) | 68.68 (19.15) | 67.08 (21.39) | 0.73 |
| Lactate, mmol/L (median, IQR) | 1.64(1.33,2.13) | 1.26(0.91,1.82) | 0.09 |
| Creatinine, mg/dL, mean (SD) | 0.31 (0.18) | 0.36 (0.83) | 0.69 |
| Blood urea nitrogen, mg/dL (median, IQR) | 9.5 (4,14) | 9.0(6,13) | 0.77 |
| Potassium, mEq/L, mean (SD) | 4.14(0.60) | 3.95 (0.71) | 0.14 |
| Sodium, mEq/L, mean (SD) | 137.24 (5.86) | 137.26 (4.35) | 0.98 |
| Magnesium, mg/dL (median, IQR) | 1.82 (1.67,2.01) | 1.82(1.67,2.01) | 0.27 |
| Phosphorus, mg/dL, mean (SD) | 4.3 (3.6,4.8) | 4.1(3.6,4.9) | 0.79 |
| Hemoglobin, g/dL, mean (SD) | 13.00 (2.64) | 13.07 (2.28) | 0.88 |
| Hematocrit %, mean (SD), mean (SD) | 39.62 (7.64) | 39.56 (7.13) | 0.96 |
| White blood, count 10^9^/L (median, IQR) | 11500 (8910,14490) | 10310 (7690,14040) | 0.16 |
| Platelets, count 10^9^/L (median, IQR) | 242000 (170000,384000) | 236000 (150000,348000) | 0.58 |
| AST, U/L (median, IQR) | 43 (29,59) | 44 (30,74) | 0.48 |
| ALT, U/L (median, IQR) | 25 (17,35) | 20 (14,59) | 0.99 |
| Total bilirubin, mg/dL (median, IQR) | 0.67(0.50,1.15) | 0.58(0.39,1.24) | 0.48 |
| Albumin, g/dL, mean (SD) | 3.44 (0.62) | 3.40 (0.65) | 0.81 |
| ***Ventilator and testing before extubation*** | | | |
| Mode of ventilator |  |  | 0.16 |
| - PS | 21 (61.76) | 128 (45.71) |  |
| - SIMV PS | 12 (35.29) | 121 (43.21) |  |
| - PC | 1 (2.94) | 31 (11.07) |  |
| ET tube size, mean (SD) | 4.04 (0.67) | 4.16 (0.68) | 0.32 |
| ET tube cuff, n (%) | 5 (14.71) | 54 (19.15) | 0.65 |
| Peak pressure, cmH_2_O, mean (SD) | 12.94 (3.77) | 12.16 (2.38) | 0.09 |
| PEEP, cmH_2_O, mean (SD) | 4.12 (0.93) | 4.04 (0.82) | 0.61 |
| Minute ventilation: V_E_ (ml/kg/min) (median, IQR) | 260(210,333.33) | 269.56(209.30,338.70) | 0.97 |
| Exhale tidal volume: V_Te,_ ml/kg, mean (SD) | 8.02 (3.63) | 8.44(4.08) | 0.63 |
| NIF test, cmH_2_O, mean (SD) | -19.68 (2.73) | -20.67(4.79) | 0.43 |

**Abbreviations:** AST: aspartate transaminase; ALT: alanine aminotransferase; ET: endotracheal tube; IQR: inter-quartile range**;** NIF: negative end expiratory pressure; PCO_2_: partial pressure of carbon dioxide; PEEP: positive end expiratory pressure; SD: standard deviation.

**Supplementary Table S2:** Sensitivity and specificity for extubation failure at each oxygen saturation cutoff point in patients with physiologic cyanosis

| **Cut point** | **Sensitivity** | **Specificity** |
| --- | --- | --- |
| $\geq$ 75 | 100 | 0 |
| $\geq$ 76 | 100 | 3.16 |
| $\geq$ 77 | 100 | 5.26 |
| $\geq$ 78 | 100 | 6.32 |
| $\geq$ 79 | 100 | 8.42 |
| $\geq$ 80 | 100 | 12.63 |
| $\geq$ 81 | 100 | 25.26 |
| $\geq$ 82 | 92.31 | 26.32 |
| $\geq$ 83 | 92.31 | 35.79 |
| $\geq$ 84 | 76.92 | 44.21 |
| $\boldsymbol{\geq}$ **85** | **76.92** | **51.58** |
| $\geq$ 86 | 61.54 | 69.47 |
| $\geq$ 87 | 61.54 | 75.79 |
| $\geq$ 88 | 61.54 | 85.26 |
| $\geq$ 89 | 46.15 | 90.53 |
| $\geq$ 90 | 30.77 | 94.74 |
| $\geq$ 91 | 23.08 | 95.79 |
| $\geq$ 92 | 15.38 | 96.84 |
| $\geq$ 93 | 15.38 | 97.89 |
| $\geq$ 94.8 | 15.38 | 98.95 |
| $\geq$ 95 | 7.69 | 98.95 |
| $\geq$ 100 | 0 | 98.95 |
| 100 | 0 | 100 |
